# Where health and environment meet: the use of invariant parameters in big data analysis

**DOI:** 10.1007/s11229-018-1844-2

**Published:** 2018-06-08

**Authors:** Sabina Leonelli, Niccolò Tempini

**Affiliations:** Exeter Centre for the Study of the Life Sciences and Department of Sociology, Anthropology and Philosophy, University of Exeter, Byrne House, St Germans Road, Exeter, EX4 4PJ UK

**Keywords:** Epidemiology, Geolocation, Data linkage, Data reuse, Inference, Data mash-ups, Localisation, Prediction, Public health

## Abstract

The use of big data to investigate the spread of infectious diseases or the impact of the built environment on human wellbeing goes beyond the realm of traditional approaches to epidemiology, and includes a large variety of data objects produced by research communities with different methods and goals. This paper addresses the conditions under which researchers link, search and interpret such diverse data by focusing on “data mash-ups”—that is the linking of data from epidemiology, biomedicine, climate and environmental science, which is typically achieved by holding one or more basic parameters, such as geolocation, as invariant. We argue that this strategy works best when epidemiologists interpret localisation procedures through an idiographic perspective that recognises their context-dependence and supports a critical evaluation of the epistemic value of geolocation data whenever they are used for new research purposes. Approaching invariants as strategic constructs can foster data linkage and re-use, and support carefully-targeted predictions in ways that can meaningfully inform public health. At the same time, it explicitly signals the limitations in the scope and applicability of the original datasets incorporated into big data collections, and thus the situated nature of data linkage exercises and their predictive power.

## Introduction

Modern epidemiology has been widely characterized as grounded on the development and application of statistical techniques to the study of the distribution and determinants of health states in human populations (Russo 2009; Broadbent 2013, p. 1). The goals of epidemiological studies are often aligned to clinical and public health objectives, thus making epidemiology one of the few scientific fields with equal footing in basic and applied research (Morabia 2005; Bauer 2008; Broadbent 2013). Philosophers interested in epidemiology have focused their attention on the use of statistical techniques, the nature of explanatory and causal claims being made, the epistemic significance of its applied goals, and the ways in which the field intersects with the social sciences, molecular biology and personalized medicine (e.g. Campaner and Galavotti 2012; Holmberg et al. 2012; Broadbent 2015; Boniolo and Nathan 2017; Russo and Vineis 2017). In this paper, we turn instead to the role and use of big data within epidemiology, and particularly the ways in which epistemic strategies to cluster and analyse big data are facilitating collaboration between epidemiology and biomedicine, environmental science and climate science.

Susanne Bauer has described epidemiological studies as “complex biopolitical assemblages, where samples, data and techniques from different contexts are temporarily brought together in particular configurations” (Bauer 2008, p. 418). Indeed, epidemiology has always been concerned with collecting and comparing datasets that are “big” in terms of their volume and variety, if not of their velocity (Kitchin 2014); and which are meant to be explored and re-used for a variety of purposes rather than solely as evidence for a given hypothesis, thus making epidemiology a quintessentially data-centric field (Leonelli 2016). By encompassing factors ranging from physiology to diet and socio-economic status of the populations in question, epidemiology has also long been concerned with the relation between information about human health and information about the lived environment (Morabia 2005). Yet, the datasets required to provide such information have typically been generated in a coordinated and centralized fashion through longitudinal projects, such as cohort or case–control studies.^1^ By subjecting whole groups to the same set of measurements and analytic tools over an extended period of time, epidemiologists developed sophisticated methods to identify and compare populations through probabilistic thinking, which in turn generated specific ways of collecting and analysing environmental data that have little in common with the methods used within climate and environmental science.^2^

The emergence of big data infrastructures, analytic tools and linkage techniques thus constitutes a novel prospect for epidemiologists interested in the intersection between health and environment. At the very least, it affects the ways in which epidemiologists identify and assemble data of relevance to their research interests. New forms of data collection and dissemination linked to advances in information and communication technologies, such as online databases, make it possible to cross-analyse and compare data that: (1) come from independent sources, including both observational and experimental data, collected via unrelated tools and for a variety of different purposes, and thus resulting in disparate data objects and formats; (2) relate to highly diverse phenomena and samples captured at widely different locations, temporalities and levels of resolution (from the genetic make-up of a specific individual to the behavioural patterns of a large population or the recurrence of a disease across generations); and (3) are produced by a wide spectrum of epistemic cultures, including groups from molecular biology, animal studies, meteorology and weather forecasting as well as patient groups, social media, hospitals and general practitioners. The digital availability of big biomedical, environmental and climate data constitutes an extraordinary opportunity for epidemiology to appropriate and analyse novel data sources concerning a large variety of environmental and health factors, thus bringing new resources to the investigation of a wide range of issues, including, for instance, the spread of infectious diseases and the impact of the built and natural environment on human wellbeing. At the same time and for the same reasons, these data lack the relative cohesion and comparability built into individual cohort or case–control studies. Their production is shaped by different understandings of disease, health and the relation between biological and environmental knowledge, as well as diverging standards for evidential reasoning and data quality evaluation. It may also be motivated by diverse goals, ranging from the testing or validation of new therapies to the development of diagnostic tools and the discovery of molecular or physiological mechanisms underpinning a given condition (Fleming et al. 2017).^3^ Finally, it is challenging to juxtapose and associate highly individualised qualitative data, such as those included in most observational studies, with the quantitative datasets acquired via high-throughput measurement instruments such as screening tools or automatic weather stations (Leonelli 2017).

This situation raises a host of philosophical questions around the epistemic warrants, methodological conditions, and potential implication of big data analysis in epidemiology. What makes it possible to link, search and interpret such data as if they constituted a single body of evidence? And how do processes of inference, extrapolation and quality assessment work, when researchers are confronted with such diverse data sources? Previous work in relation to the life sciences and biomedicine illustrated how exploring these questions involves in-depth study of data practices and related theoretical and methodological commitments (O’Malley and Soyer 2012; Bechtel 2013; Plutynski 2013; Leonelli 2016). In this paper, we propose to bring these questions to bear on the case of “data mash-ups,” which cluster data coming from epidemiology, biomedicine, and climate and environmental science. In the words of their developers and users, data mash-ups are “a dynamic, explorative and ongoing exercise of processing, mixing and analysing different types of data together to produce a unified and unique output which can be potentially more useful than and accessed independently of the original individual datasets” (Daniel and Matera 2014). We shall focus on a specific strategy used to produce data mash-ups. This is to combine data extracted from various digital repositories by clustering the data around a basic parameter of choice, which takes on the role of an invariant point of reference. Geolocation data are often used as an invariant in this way, as it seems relatively straightforward to align data points collected on the same location in order to assess potential correlations. We are interested in uncovering the conditions under which the use of geolocation as an invariance strategy can fruitfully serve efforts to analyse and interpret big data assemblages; and the advantages and disadvantages involved in adopting this strategy.^4^

Building on Shavit and Griesemer’s work on notions of locality employed in the making and validation of biodiversity surveys (2009), we will argue that this strategy is most effective when epidemiologists use an *idiographic* conceptualisation of geolocation, which recognises the extent to which locality measurements depend on the investigators’ instruments and perception; and the “interactive construction” of space by, on the one hand, the very act of data collection, and on the other hand, the way in which living systems themselves, which are the object of an observation and data collection exercise, relationally shape the environment they live in.^5^ This view of geolocation calls attention to the situated nature of the processes through which particular phenomena are given spatial references (which we shall refer to as localisation processes). As we shall argue, this in turn highlights the epistemic importance of being able to consult information about data provenance when assessing the quality and evidential value of geolocation measurements, and being able to disaggregate data clusters—such as mash-ups—when this is required to verify the reliability of specific data interpretations.

The explicit adoption of an idiographic interpretation of geolocation can thus foster data linkage and re-use, while also signposting the limitations in the scope and applicability of the original datasets that are incorporated into big data collections. This approach to localisation can also support carefully-targeted generalizations and predictions, hence playing an important role in informing decision-making concerning public health. Indeed, we shall argue that conceptualising localisation in this way fosters critical consideration of the *scope* and *targets* of generalizations and predictions derived from the analysis of data clusters.

Our discussion is grounded on the study of one specific experience in data mash-ups, namely the development of the Medical and Environmental Data Mash-Up Infrastructure—devised by UK-based epidemiologists in collaboration with climate and environmental scientists in order to enable extensive data linkage, thus providing a platform for the performance of data mash-ups.^6^ Our analysis will proceed as follows. In the first section of the paper, we introduce MEDMI and provide examples of how it has enabled the production and analysis of data mash-ups. In Sect. 3, we discuss the notion of invariance, how geolocation is used as an invariant parameter in MEDMI-enabled data mash-ups, and with which results. Section 4 discusses two epistemological problems that the use of geolocation as an invariant point of reference needs to overcome: first, geolocation is not actually invariant across the datasets in question; and second, geolocation data need to be re-situated in order to be intelligible to researchers who wish to use them. In Sect. 5, we tackle these two concerns by proposing to view invariants as strategic constructs. In Sect. 6 we then argue that conceptualising locality as idiographic helps to highlight how invariance is not something “found” by trawling through datasets, but rather is construed through the calibration of locational data with background assumptions and theories around how the phenomena in question interact with their environment. As we illustrate in Sect. 7, this conceptualization helps to identify the epistemic advantages of using geolocation as invariant, and the reasons why researchers regard it as fruitful despite the concerns presented in Sect. 4. In closing, we draw a general lesson from our analysis of data mash-ups: using invariance strategies for big data integration across health and the environment works best when the invariance is contextualised and data aggregates can be disaggregated for ease of re-interpretation and quality control.

## Health/environment data mash-ups: the case of MEDMI

The Medical and Environmental Data Mashup Infrastructure is a project aiming to facilitate research-grade mash-ups of weather, environmental, demographic and human health data. MEDMI was developed by a consortium of four main partners (University of Exeter Medical School, London School of Hygiene and Tropical Medicine, UK Met Office and Public Health England) over a 3-year period (2013–2016), with funding from the Medical Research Council and the Natural and Environmental Research Council. It was conceived as a novel type of infrastructural resource geared towards innovative interdisciplinary science. MEDMI developers named the project a mash-up as it “implies easy and fast integration of different types and sources of data, frequently using open application programming interfaces and data sources, to produce enriched results that were not necessarily the original reason for assembling the raw source data” (Fleming et al. 2014, p. 1730).^7^ The initial focus was the assemblage of data relevant to constructing time series analyses of the seasonality of specific UK pathogens and related diseases. To this aim, MEDMI aimed to combine data relating to:

- territory (including information about the landscape, levels of urbanization and population density, as well as socio-economic data such as education levels and average income, typically organized by municipality and region of residence);
- climate (including data about levels of rainfall and humidity, temperature and altitude, which provide insight into the weather conditions at different locations);
- patients (including symptoms recorded by general practitioners, and rate of incidence, time and severity of diseases); and
- pathogen biology (including information about the physiology, life cycle and nutrition of the microbes in question, which helps to estimate the conditions under which the pathogen is likely to thrive and be most damaging to hosts).^8^

Gathering data of such different types and origin into a single infrastructure promises to open new avenues of research, with interdisciplinary investigations on the links between the environment and human health acquiring a bigger scale; and executing more ambitious research designs than was possible before.

This is significant given the considerable disciplinary barriers traditionally separating these data types and sources from each other. Data gathered in epidemiological cohort studies may well include data on the environment, climate and landscape, but this typically happens in the context of specific projects rather than through linkage to existing (and much more comprehensive) sources of weather and environmental data, such as the MET Office. An infrastructure such as MEDMI endeavours to offer researchers a simple point of access to several data collections, and the tools and resources to be able to parse, compare, extract and analyse the data safely and reliably, thus enhancing the robustness and validity of the research by enabling researchers to triangulate between different types of findings. To realize this vision, MEDMI developers divided the project into three components: the sourcing of datasets; the development of methods and tools to perform the “mashing up” and enable new analyses of putative links between environment and human health; and the demonstration of methods and tools via pilot research pieces, which would showcase what MEDMI affords to research.

In the analysis that follows, we focus on one of the three demonstrative research pieces, with an interest in exemplifying and understanding the epistemic strategy of invariance construction through which it becomes possible to link, compare and juxtapose datasets that have different origins and focus.

## Localisation as an invariance strategy

An invariant is typically defined by methodology textbooks as an object or a quantity that remains unchanged under a group of transformations.^9^ While the concept of invariant finds its origin in mathematics (with a subfield fully devoted to invariant theory), it has been adopted across several disciplines by widening the range of objects and properties that can be considered as invariant. Within experimental science, positing a given set of parameters as invariant can provide a stable reference point against which experimental variables of interest can be more easily detected and hypotheses tested. Similarly, finding invariant criteria for grouping data has long constituted a strategy for researchers interested in analysing large datasets through statistical methods, particularly when such datasets include observational data. Invariant parameters are invaluable resources for clustering and aligning highly disorganised and heterogeneous data collections, and it is no surprise that the toolset used by epidemiologists interested in mashing up medical, environmental and climate data prominently features techniques for identifying and controlling them.

Perhaps the parameters most widely used as invariant for big data analysis are those relating to geolocation, by which we mean any measurement or estimate of the geographical location of an object—for instance, as achieved through reference to longitudinal/latitudinal information, postal address, global positioning system (GPS) coordinates, or other satellite measurements. Using geolocation parameters as invariants was a key strategy in one of the most successful uses of MEDMI to date, which investigated the seasonality of a set of common pathogens including the *Campylobacter* bacterium, well-known to UK public health officials as a common agent of food poisoning. In this study researchers compared existing data on reported cases of *Campylobacter* infection at particular locations to weather temperature data recorded on those locations in the days before the cases of infection were first identified. The outcome was a clear correlation between higher temperatures and incidence of pathogen activity (Djennad et al. 2017).

This was not a ground-breaking result per se. The conditions under which the bacterium is likely to infect humans were already known to be susceptible to temperature, and the study therefore confirmed a highly plausible hypothesis. What was more interesting for researchers was the usefulness of this study as a validation of their general approach—in other words, as a demonstration that mashing up climatic and health datasets by positing geolocation as an invariant could provide reliable and independently confirmed knowledge on disease seasonality across a wide range of environments. Scaling up from this proof-of-concept, researchers went on to apply the same strategy to a much larger dataset, which included extensive weather and environmental data, as well as infection case data for over 2000 named species or serotypes of pathogens as reflected in 14 million records of lab analyses conducted over a period of 25 years. This data mash-up, called LabBase2 Trawl, was organised in similar ways to the *Campylobacter* study, and yet the audacity of its scale and scope made its results much more interesting and insightful, not least because they could not have been obtained through more traditional epidemiological work. Among other things, researchers could now compare the seasonality of different strands of the same pathogen, relate shifts in seasonal behaviours to specific weather patterns, and assess the incidence across the population in terms of age distribution, socio-economic status and weather exposure.

At its most ambitious, this kind of data mash-ups could provide evidence to model the impact of climate change on infectious disease incidence and seasonality. This would be a significant achievement for a study grounded on the deceptively simple assumption that highly diverse data can be correlated through the location in which they were originally collected. The potential of these findings to inform decisions relating to public health and the management of health services in the future makes it particularly important to ensure that the assumptions and methods underpinning the research are sound and reliable.

## Problems with the use of geolocation as invariant

While the use of geolocation as invariant was crucial to the production of informative and novel knowledge claims in the LabTrawl2 study, this strategy for the performance of data mash-ups is susceptible to some major challenges. In this section, we discuss two concerns which have major epistemic implications in terms of the reliability and trustworthiness of the knowledge claims acquired through this approach: (1) geolocation measurements *are not actually invariant*, with each measurement referring to a specific understanding of location and thus, potentially, a different place altogether; and (2) *despite* the adjustments made to the datasets in order to create the mash-up, the reliability of the data for the specific investigative purposes of any one project cannot be taken for granted, and indeed researchers wishing to re-use data within new projects need to spend time assessing whether the data are fit for purpose and investigating which interpretations of the data make sense in light of their provenance.

Problem (1) calls into question the very validity of assuming geolocation as an invariant property around which evidence for the study is assembled and built. This is easily uncovered when considering that the datasets collected in studies like the *Campylobacter* project and LabBase2 Trawl employ very different scales and assumptions for geolocation measurements. Some of the climate data are ordered on weather grids to establish spatial coordinates, while others employ high resolution GPS coordinates. Environmental and population data (such as those collected by the national census) are sometimes geolocated with reference to a given geographical radius (say, 25 km), and other times to administrative standards such as postcodes, constituencies or districts. These are quite literally different definitions of space, and they are difficult to correlate with each other. Each approach is also likely to have different criteria for what is worth measuring in the first place (as in the case of census data for instance, where locations are defined by homogeneous clusters of inhabitants and can cover areas of very different physical size), thus providing a very uneven sample when data collected through different criteria of exclusion are merged.

Even trickier is the issue of identifying locations for human health incidents. These locations are crucial to virtually any study performed through MEDMI, as these studies—like those discussed above—aim to improve public health provision. Nevertheless, MEDMI is unable to use location information about patients, such as their house address, since these constitute identifying information and can only be shared under conditions of confidentiality which MEDMI cannot guarantee.^10^ LabTrawl2 researchers therefore used the address of labs that process patient samples as proxies for the geolocation of the patients themselves, based on the assumption that patients are most likely to use a lab close to where they live (in their words, these addresses signal “catchment areas” for individual patients). The shortcomings of this assumption are obvious, ranging from the possibility that patients did not in fact use the lab closest to their place of residence, to the problem of rural regions where catchment areas for any one lab are likely to be very extensive—not to mention potential time lags and people contracting illnesses while travelling.

Furthermore, not only the spatial definition of the geolocation data, but also the ways in which their values are obtained are highly diverse, generating results that are not always consistent or well documented. In the following quote, an epidemiologist discusses the problems involved in extracting relevant data from the weather datasets:

“The Met Office holds weather parameters that vary from changes per minute to changes per year, or decade, so it has just a vast amount of data, and extracting it in exactly the right format is more difficult than it seems. Some of the datasets are held in the format that’s relatively easy to extract, and some *there’s no published data on the time scale and resolution that we want*. The way that the weather data is held… You have ground stations, and the ground stations record the weather, and so there’s gaps between the individual ground stations, and the different parameters measured in different ways. So, they must do interpolations to estimate the area between the ground stations and then produce the data in the format that they want it. So some things which I would have thought would have been easily collectible, like sunlight, they only produce on a weekly, monthly basis—it’s monthly, I think—so we haven’t been able to get daily or weekly sunshine recordings. It’s the same for a whole lot of other parameters. It’s trying to get the exact parameters we want, that’s more difficult. I think it’s to do with the ease, or lack of, being able to extract it” (Researcher 35)

Such variability makes it hard to control for error and sampling biases; and makes it necessary for researchers to use calculations (in the words of researcher 35, “interpolations”) involving various kinds of judgements made to estimate data quality and reliability. The widespread awareness that geolocation data are not necessarily comparable is confronted and remedied through tailored operations of data evaluation and processing, aimed at making the data comparable and supporting their alignment.

Things become even more complex when considering the full diversity of data involved in data mash-ups, as eloquently explained by another MEDMI participant:

“So putting all that together and trying to summarise the problems, *you have data at lots of different scales of spatial aggregation and at the same time sometimes lots of different scales of time resolution as well*—some of the data is daily, like the weather data could be daily, the health data is monthly, it could be annual in some cases, and then the Census data, of course, is… well it’s annual, I suppose, by the time you put the predictions in there, but it’s really ten-yearly [laughs]. And *if you are going to try and provide a platform which kind of integrates all of this data, what is your baseline? I mean, what is your baseline?”* (Researcher 28, our emphasis)

This brings us to problem (2). Taken literally, the above quote could be interpreted as a strong objection to the validity and epistemic usefulness of data mash-ups. The researcher is implying that in a situation such as MEDMI, where the scales of spatial aggregation and temporal resolution in the datasets are so diverse, there is simply *no generalizable way* of setting a baseline for data linkage. It is tempting to read this as a profoundly sceptical argument about the epistemic value of data-linking practices, which demonstrates that large data assemblages created without regard for the specific characteristics of each dataset are intrinsically unreliable and potentially misleading. This sceptical view is very important to articulate and explicitly discuss, particularly given the claims made by some big data analysts around the extent to which increasing the volume and diversity of data can offset epistemic concerns around the accuracy and relevance of data as evidence within causal reasoning (e.g. Mayer-Schönberger and Cukier 2013).^11^ The two problems with the use of geolocation as invariants which we identified in this section point to the opposite conclusion: namely, that the accumulation and linkage of large and diverse datasets can be confusing at best and treacherous at worst, pushing researchers to forgo crucial information about the circumstances in which data have been collected and curated, and thus underestimate existing discrepancies in the meaning and scales of the parameters employed. This in turn may result in researchers becoming insensitive to the specific aims and conditions of data collection, and disregarding information that may well prove crucial to an adequate contextualisation and interpretation of the data (a situation that would occur, for instance, if anybody used MEDMI to link health and weather data without considering their different temporal scales).

## Confronting the problems: invariants as situated constructs

There is however another way to interpret the concern voiced by researcher 28, which similarly captures epistemic worries about potential misuses of big data linkage, while at the same time salvaging and explaining the value of such efforts towards producing reliable forms of knowledge. This interpretation reads the researcher’s worry as concerning *the conditions under which* researchers decide the parameters for data linkage and integration. While it is true that there is no baseline for data linkage that can work for any situation of data re-use, *it may be possible*—*indeed, necessary*—*to identify the best baseline for data linkage within any one project* by taking the specific aims, methods and circumstances of data analysis into account. Indeed, MEDMI researcher 28 went on to explain that the difficulties with timelags were resolved by letting go of the idea of finding a generalised baseline altogether (thus avoiding, in the researcher’s own words, the creation of “a set of gridded data sitting there that you’ve artificially produced”). What was decided instead was to encourage MEDMI users to find the best invariant for each and every project, and to do it in ways that would help them to assess which data are actually relevant and adequate for their line of investigation. For instance, for a project investigating the relation between the number of asthma diagnoses and levels of humidity in the atmosphere at a given location, researchers settled on the time resolution of health data as the baseline around which all other data would be evaluated and compared—resulting in weather data being mined only for monthly averages rather than daily measurements. By abandoning any pretence of working with a perfect dataset, this strategy takes on board a lesson that clearly emerges from the history of science, and most prominently the historical sciences: imperfect and imprecise datasets can be successfully used as evidence towards important discoveries, as long as their limitations are clearly acknowledged and incorporated into the analysis (e.g. Chapman and Wylie 2016 and Currie 2017).

“Finding one’ baseline” thus captures the requirement that the assumptions made by researchers about the status of the datasets—and in particular, the classifications of location that they are built on—need to be checked at every step of data re-use, to make sure that the ways in which datasets are being (re)interpreted, compared and juxtaposed with one another is still consistent and supported by the available data.^12^ Within this view, big data linkage has epistemic value only when researchers are willing and able to adapt the parameters through which the invariant point of reference is constructed to their situated assessment of what is plausible within the specific inquiry at hand. In turn, the ability to identify and establish the right baseline for any given project stems from the ability to check the original sources used in data mash-ups, so as to tailor the analysis and interpretation of the data to the assumptions made in data collection or subsequent processing. Assumptions thought to be irrelevant or unimportant at an early stage of the mash-up process, such as those concerning the temporal and spatial scales of datasets, may turn out to be crucial, at a later stage, to the re-use of data in a new context. Hence making those assumptions explicit and easy to track is essential for researchers interested in using the data as evidence.

One MEDMI researcher gave a useful illustration of this point, while discussing the difficulties of formulating causal claims on the basis of highly de-contextualised data:

“You can’t necessarily look at lags or manipulate the data if it’s all linked. So, for example, if you have a flood, people can be killed right away or they can be harmed from that exposure. For example, their mental health, it doesn’t actually give evidence of that for months. So there’s lags from exposure to health onsets. If you link the data ahead of time you’ve almost set the conditions for what that lag is, you’ve defined that lag. If you want people to really use the data, explore the data, you can’t do that.” (Researcher 34)

The researcher is pointing to the problems encountered when re-deploying data mash-ups beyond the original situation in which they were developed, without re-examining the assumptions built into the ways such datasets are linked. The example she gives concerns the analysis of data to determine the impact of a flood on people’s mental health. An initial assessment of the impact may involve linking data on hospital admissions to data coming from social services and charities, who would be in charge of assisting individuals in the immediate aftermath of such an event. A different problem is trying to assess the impact of the flood in the long-term, once individuals have been exposed to the consequences of the flood for their livelihoods and communities. Investigating the impact of the flood on health certainly involves linking several datasets, and yet the ways in which those are linked—and the assumptions made about which units of space and time are most useful as an invariant—may change dramatically as the situation unfolds (for instance, if there is reason to believe that the mental health of the population in question is declining long after the event itself). The researcher therefore points out that it is crucial for the assumptions made when linking data to be re-examined regularly, in order to check whether the chosen parameters are still relevant and credible, and whether they are up to date vis-à-vis the influx of new data and changes in the knowledge landscape within which the research takes place.

This example highlights how the construction of an invariant point of reference through which datasets can be juxtaposed with one another cannot be achieved through fixed, stable solutions dictated solely by the formats through which location is classified. Using a parameter as invariant needs contingent and situated evaluation of alternative paths of action in relation to the research question and the theoretical scaffolding supporting the investigation. These are the conditions for the *construction* of an invariant, which are not inherent in datasets that have completely different origins. Infrastructures such as MEDMI are developed in order to systematically help to establish and satisfy these conditions.

## Mashing up and separating out: the idiographic approach to geolocation

In the previous section, we have argued that the use of geolocation as an invariant parameter for data linkage is plagued by serious epistemic concerns, and yet there is a way to justify the value of this approach—and thus explain the emergence of data mashups within and beyond contemporary epidemiology, and the broader trends towards combining and repurposing increasingly diverse data sources. This is to regard the identification and use of invariant parameters as an activity firmly situated within—and adapted to—specific contexts of inquiry. In this section, we discuss a way to conceptualise the notion of geolocation (which we have thus far used solely as an actor’s category), which usefully aligns with our view of invariants as situated constructs.

Our starting point is a distinction between two views on locality discussed in Shavit and Griesemer (2009). The first is the *nomothetic* approach, which is construed by applying a priori principles to the collection and analysis of spatial measurements. It is linked to what Shavit and Griesemer call “exogenous” perspective on space, whereby space is presented as a context-independent dimension separate from the observers, the circumstances of measurement, and the phenomena of interest. This mode of dividing and recording space starts from the mathematical assumption that the units that mark space are quantitative distances. The nomothetic conceptualisation of locality thus understands change in location as homogeneous, proportional and independent from other variables, and is typically grounded on law-like generalisations and statistical approaches to sampling. Many of the ways in which information about location is extracted from satellite measurements are grounded in this perspective.

The second conceptualisation of locality is the *idiographic* approach, which is construed in relation to the specific circumstances of inquiry. Shavit and Griesemer present the idiographic perspective on space as “system-interactive”, in the sense of taking into account what observers know about the phenomena of interest as well as the procedures and instruments used for measurement. Here researchers’ background knowledge about the *kinds* of space inhabited by the phenomena of interest is strategically employed. Space is marked through an expert, contextual judgement about the relevance of a particular parameter to the environment being studied, relative to the phenomena of interest (e.g. water streams are a locality where otter activity is more likely). The idiographic conceptualisation of locality thus understands change in location as inhomogeneous, discontinuous and relative to other variables, and focuses on “particular, unique cases and circumstances” (Shavit and Griesemer 2009, p. 284).

The distinction between exogenous and system-interactive approaches to space has immediate implications for the value attributed to location data, and the conditions under which they are re-purposed and interpreted. Conceptualising space as exogenous pushes researchers to assume that location measurements (such as against grids such as GPS or latitudinal/longitudinal coordinates) are more valuable than other contextual data, since these data are seen to reliably identify locality independently of other factors. On the contrary, a system-interactive notion of space encourages researchers to actively question whether and how any available location measurements can support the inquiry at hand. This aligns neatly with our claim that invariant parameters need to be identified and used in ways that are tailored to the specific circumstance of inquiry. The idiographic conceptualisation of locality usefully complements our characterisation of the use of geolocation as invariant, and enables us to explore in more detail some of the epistemological consequences of this position for the use of big data in epidemiology.

Indeed, Shavit and Griesemer highlight the epistemic risks involved in adopting an exogenous notions of space, which include underestimating the significant variation characterising biologists’ understanding of the environments and organisms captured by biodiversity surveys. Similarly, we argue that researchers who use geolocation as an invariant parameter for big data analysis should conceptualise location as idiographic, so as to avoid the risk of underestimating the extent to which assumptions intrinsic to different sets of measurements affect how data are linked and interpreted. The ways in which different datasets are linked need to reflect the researchers’ understanding of the circumstances in which data were produced and the assumptions that researchers hold about specific spatial units (e.g. what are their relevant qualities, and how a unit can relate to differently drawn spatial units from different source datasets).

This position is perfectly compatible with the recognition that the nomothetic approach is indispensable to the construction of highly standardised and immediately comparable systems of variables and measurements, which underpin data collection efforts such as the production of weather maps. What we wish to stress is that a nomothetic conceptualisation of geolocation is not as helpful to the linkage of highly heterogeneous datasets. Far from being a nomothetic entity, locality in MEDMI data mash-ups is construed on the basis of data availability, causal understandings of relations among relevant phenomena, familiarity with the territory at hand, and specific goals of inquiry. Find a baseline for data linkage, as in the case of researcher 28 discussed in Sects. 4 and 5, involves considering all of these elements. Another way to stress this point is to ask what is actually being localized in MEDMI—what is the object of the analyses and operations of dataset juxtaposition that researchers carry out. Arguably, what is being constructed as invariant parameter is the space in which a particular *set of relations* between pathogens, humans and environment unfolded at a given time.

This conceptualisation helps to make better sense of MEDMI researchers’ inability to identify a straightforward approach to the task of comparing and relating geolocation data from different datasets, as exemplified above. For a causal claim derived from correlations in a data mash-up to be epistemically robust, its reliability needs to be checked against relevant metadata documenting the circumstances of their collection and subsequent processing. Data need to be re-contextualised as required to support the specific assumptions and context of their re-use, and fit the questions and phenomena under consideration. As Leonelli discussed in relation to research in the life sciences, using data as evidence for claims involves situating the history of a given dataset in relation to the inquiry at hand (Leonelli 2016). It follows that when data are evaluated within a new inquiry and in relation to a new question, researchers need to critically re-examine assumptions made around the evidential value of data, including how geolocation has been construed since the generation of the datasets in question and assumptions around what can work as an invariant parameter. Hence when asking about the causal links between microbial environments, climate and human health, it is not possible to directly juxtapose climate, patient and pathogen geolocation data in the format dictated by the repositories in which these data are stored. Researchers need to assess the extent to which the history of those data aligns with their own interests and background knowledge.

To understand the implications of this insight for the epistemic role of data mash-ups, let us consider again the definition of data mash-ups provided at the start of this paper: “a unified and unique output which can be potentially more useful than and accessed independently of the original individual datasets.” This formulation could be misconstrued as implying that the original datasets absorbed into a data mash-up (and their histories) no longer matter. In other words, it could be read to endorse an exogenous view of space as sufficient to ground inference from data mash-ups. By contrast, we argue that the credibility and reliability of data mash-up as research tools hinges on adopting a system-interactive view of space, and thus an idiographic approach to geolocation data. In our view, the most important term in the above definition of mash-ups is “potentially”, which indicates that the epistemic value of any data aggregate will always depend on researchers’ ability to evaluate whether the spatial and temporal assumptions made within each datasets are compatible with each other and with the goals and commitments of the study at hand (Aaltonen and Tempini 2014). The histories of original datasets continue to matter throughout their journeys, regardless of how they are assembled and visualised in clustering exercises such as data mash-ups.

## The epistemic advantages of idiographic approaches to invariants

The idiographic approach to geolocation highlights the necessarily limited scope and applicability of the studies centred on the reuse of big data collections, and the diversity of theoretical and methodological commitments potentially underlying location measurements. Given these limitations and the considerable epistemic concerns raised in Sect. 4, it is tempting to argue that the construction of data mash-ups based on assuming geolocation data as invariant is epistemically indefensible, insofar as it creates potentially unreliable correlations that do not account for the biases and assumptions that shape the collection of the geolocation data. This is not however the lesson that we draw from our analysis. On the contrary, we claim that researchers can use the challenges involved in aligning and interpreting different types of geolocation data to their advantage, by explicitly considering whether and how the choice to use a given parameter as invariant can support their research goals and interpretation of the data. Working with idiographic concepts of locality can be enormously helpful in this respect, because it forces researchers to reflect on assumptions made within the data and the extent to which those assumptions fit existing understandings of the phenomena under study. A nomothetic conceptualisation of locality continues to be extremely valuable at the stage of data collection, where it helps to acquire consistent and usable datasets. At the stage of data linkage and analysis, by contrast, the idiographic view is preferable since it helps researchers to critically evaluate the principles underpinning data collection, and choosing methods in ways that are tailored to their changing goals and easily amenable to further scrutiny. The same argument holds for other variables typically used as invariants in data mash-ups. The most obvious example is time, which could be similarly conceptualised as exogenous or system-interactive, resulting in comparable issues when attempting to link and mine diverse temporal datasets.

A project like MEDMI constitutes an excellent instance of how big data research practices, standards and infrastructures can be designed to *exploit* the idiographic nature of geolocation and other properties taken as invariant, rather than trying to hide it away in an effort to support an exogenous view. This involves investing effort in documenting practices of data manipulation and processing, and the ways in which decisions taken during these phases of research relate to the goals and circumstances of the study at hand, as well as the assumptions and background knowledge characterising the field and research community in question.^13^ Such efforts enhance researchers’ ability to explore correlations and datasets that were not brought together before, thus enrolling additional evidence towards the identification of causal links between environmental, biological and medical factors. Most importantly, this can happen in new combinations of scale, enabling studies of wider scope and range than before, and better resolution. In the words of a MEDMI researcher:

“The fact that you’ve got cases where you’ve got a mixture of geography and time means that when you apply something like temperature to that, you’ve got three different parameters, and you can look much more closely at how those parameters interact. The seasonal distribution of cases, for example, has an element which is probably related to the weather parameters, but you can separate it out geographically, because the temperature differs across the country. So if you apply it in that way, you can learn more than if you just used the averaged data. So I think *we’ve demonstrated that local data linkage has a strong potential for answering some of the questions which weren’t very easily answered by using just averaged data*. It’s providing a greater resolution, and ability to tease out the weather parameters from the seasonal and the geographic.” (Researcher 35)

Additionally, the opportunity to consider health and environment as part of the same studies can affect how researchers define key concepts such as wellbeing, illness and exposure, model and prevent health concerns, and intervene on emerging threats. This may involve fostering an integrated approach to the ontology of disease as involving not only mechanisms “internal” to pathogen/host interactions, but also the wider environment in which pathogen and host develop. It may also transform the ways in which researchers understand pathogen behaviour and ecology, including the diversity of its interactions with other organisms. Arguably, new forms of data linkage are instigating a move away from the idea of obligate pathogen, which are modelled as causes of disease no matter the circumstances, and towards a more dynamic understanding of health and disease as functions of the relation between organisms and their microbiome—a view which reflects the latest insights on pathogenicity coming from the life sciences (Casadevall and Pirofski 2014).^14^ This may in turn lead to new insights concerning the relevant sources of variability and triggers for virulence among microorganisms, and the conditions for and modalities of public health interventions.

Furthermore, there are significant methodological and organisational advantages to be gained from the considerate use of invariance strategies for data linkage. One is the development of new ways to combine and manage expertise across different fields and research traditions, and thus new forms of interdisciplinarity explicitly targeted to the resolution of societal challenges or conceptual problems. Another is the development of models, tools and algorithms of wider applicability, which could benefit research groups beyond those engaged in any one data mash-up. A case in point is the continuing insistence by MEDMI researchers to research the epistemic implications of the imprecision inherent in the geolocation data used for the LabTrawl2 study. There are ongoing efforts to test how the use of lab catchment areas as a spatial proxy for the location of patients affects the conclusions of the study, for instance by comparing the correlations obtained when using lab postcodes with those generated when the postcodes of the patients were available (Djennad et al. 2018). This is an attempt to understand whether there were systematic discrepancies, thus improving the accuracy of the predictions derived from data analysis and clarifying the circumstances under which those predictions are more likely to be reliable.

This example points to one final feature of the idiographic approach to invariants that we wish to highlight, which is the potential it carries to improve the predictive power of models produced from big data analyses. Developing such models is a coveted goal for epidemiological studies, because these tools can be invaluable in informing decision-making relating to public health. It goes without saying, for example, that developing a model able to accurately and reliably predict the locations and timings of outbreaks of respiratory diseases in the UK would be an enormous scientific and social achievement, with direct impact on how hospitals and GPs are resourced throughout the year. Prima facie, it could be argued that the drive to develop general models favours the adoption of the nomothetic approach to invariants such as geolocation, since this highly general, non-local approach is well-suited to developing predictive models with a wide scope of applicability. And yet, there is no guarantee that such models would be accurate and reliable in their predictions, regardless of their field of application. This is precisely the problem that adopting an explicitly idiographic approach to invariants can solve. From that perspective, the applicability of a predictive model is not assumed a priori, but rather is actively questioned and investigated as part of the research programme. As in the above case of LabTrawl2, researchers who recognise the situated nature of data linkage have an incentive to continue to interrogate the ways in which data are integrated within the model, thus obtaining more knowledge about the circumstances under which the model can be trusted.

## Conclusion: on the use of invariance strategies for big data analysis

We have argued that an idiographic conceptualisation of invariants used in big data linkage recognises the importance of data provenance, facilitates the contextual evaluation of data and helps to assess the reliability of specific interpretations. The adoption of an idiographic interpretation of geolocation can thus foster effective data re-use, while also signalling the limitations in the scope and applicability of the original studies incorporated into big data collections, and thus the situated nature of data clustering exercises such as the mash-ups.

In our analysis we touched only briefly on a key potential objection to our argument, which is that it may seem to dismiss the epistemic value of nomothetic approaches to localisation. This is an important concern given the crucial role played by standards, conventions and norms in designing data collection and ordering procedures and facilitating the comparison of data collected across different locations. A good example of this are weather grids, which are based on a priori decisions around the expected resolution, format and computability of the resulting data. Indeed, it is sometimes argued that big data linkage and analysis can only happen when a priori principles for data collection, formatting and dissemination are agreed and acted upon. We do not disagree on the significance of nomothetic approaches to localisation, particularly given what Griesemer and Shavit describe as the “inherent ambiguity of locality” (2009) and the importance of finding ways to managing it. There is no doubt that nomothetic approaches are significant to localisation procedures and have numerous advantages when attempting to construe comparable datasets and longitudinal studies. What we wish to highlight are the problems involved in relying on a nomothetic view whenever linking geolocation data from many different sources, each of which is likely to be using different norms around what constitutes geolocation and how it is measured. In those cases, a nomothetic approach does not help researchers to critically evaluate the implications of mashing up the data and the reliability of the inferences being drawn on that evidential basis.

The case of geolocation is comparable to those of other parameters routinely used as invariant within data mash-ups, such as seasonality, temporal coordinates, random noise and similarity in patient responses to pathogens. The data used to capture these variables are as variable and unstable as geolocation data; and their linkage can involve even more complex interpolations. At the same time, embracing the situated nature of data collection and re-use can help researchers to conduct mash-up studies in ways that harness the scale and scope offered by big data analysis, while retaining the sensitivity to the epistemic relevance of specific contexts and samples that has long been characteristic of epidemiology. This does not involve giving up on predictive models of wide-ranging applicability. What it does involve, instead, is abandoning the idea that such models can be judged to be valid and trustworthy regardless of their context of application. As we argued, the history and context of the data may affect whether or not they constitute credible evidence—and thus affect the reliability of the predictive inferences extracted from data mash-ups. Acknowledging this situation helps to foster a welcome critical attitude among researchers towards the scope of any given prediction. Where and when to trust a predictive model becomes a matter for empirical scrutiny, which may well include a second look at the original data used to inform inferential reasoning. Successful predictive models can provide a platform for public health decision-making, but not by virtue of relying on a universal, nomothetic conception of geolocation or other invariants: rather, their success should be related to their capacity to fit a wide variety of situations—a capacity whose limits need to be probed and assessed in order to improve the reliability of the model.

We conclude that using basic parameters such as those relating to geolocation as invariance strategies can be effective for data integration across diverse sources and areas of expertise. It affects how epidemiologists understand and model the ontology of disease, the nature of pathogenicity and the dynamic relations between micro-organisms, hosts and the environment. It also influences the conceptual directions, methods and tools adopted within the field, and the ways in which epidemiology intersects with skills, results and insights coming from other research areas. Whether the strategy is methodologically and epistemically reliable depends on the care with which the researchers involved in developing and analysing data mash-ups keep track of—and continue to probe—the limitations and constraints to data analysis and the provenance, history and processing of the data being linked together.

## Footnotes

1 Technically in epidemiology only cohort studies are considered to be longitudinal, however often case control studies are longitudinal in practice.

2 See Morabia (2005) for an overview of the different types of study designs used to assemble evidence and test hypotheses.

3 See also Knorr-Cetina (1999), Leonelli (2012) and Longino (2013) for analyses of how diverse and sometimes incompatible conceptual and methodological assumptions can affect data sharing and re-use across different epistemic cultures.

4 Our discussion focuses on the spatial coordinates involved in geolocation, and thus on conceptualizations of space and locality. As we note in our conclusion, temporal coordinates are as important as spatial coordinates for geolocation, and our argument is also meant to apply to temporal data. More research on the peculiarities of temporal data is required to examine the implications of this point.

5 Shavit and Griesemer elaborate on the distinction between idiographic and nomothetic originally proposed by German philosopher Windelband ([1894] 1980). A detailed investigation of the history of these concepts and their role in Windelband’s work would be instructive, but is not directly relevant to our arguments here, which build primarily on Shavit and Griesemer’s analysis of the research practices privileged by those who collect and interpret biological and environmental data.

6 Our analysis is empirically grounded on the study of MEDMI publications and other materials available on their website (MEDMI website 2017); and on collaborative work carried out with MEDMI staff between April 2015 and January 2017, which included interviewing 24 MEDMI participants around their experiences in the project. The quotes used in this paper are taken from transcripts of these interviews. Those transcripts which participants have agreed to publicly disclose are openly accessible within the Exeter Data Studies data collection hosted by Zenodo (URL: https://zenodo.org/communities/datastudies/?page=1&size=20).

7 The term mash-up is popular in the web development world to refer to interfaces that offer integrated and seamless functionality, despite sourcing data from multiple storage locations; and to this extent it has been often used to refer to applications involving mapping software or locational data.

8 In practice, MEDMI encountered challenges in incorporating some of these data sources and particularly data from patients. At the moment of writing, the only medical data available are lab reports concerning infectious disease and death data for a limited number of authorized researchers. For a detailed discussion of data access and security in MEDMI, see Tempini and Leonelli (2018).

9 For example Weisstein (2017) and Popov (2017).

10 MEDMI lacks strong measures to prevent re-identification (Tempini and Leonelli, 2018).

11 For critiques of this view, see Leonelli (2014) and Calude and Longo (2016).

12 As pointed out by Edwards (2010) and Bokulich (2018), among others, models often play a crucial role in this process.

13 Metadata that are stripped away from the original dataset for a particular purpose may prove essential when the goals and methods of inquiry change (Leonelli 2016).

14 We thank an anonymous reviewer who pushed us on this point, which deserves much more careful scrutiny than what is possible within the scope of this paper.
